# Society Meetings

**Published:** 1904-12

**Authors:** 


### SOCIETY MEETINGS.

---

The Southern Surgical and Gynecological Association will hold its seventeenth annual meeting at the Hotel Hillman, Birmingham, Ala., December 13-15, 1904, under the following mentioned administration: president, Floyd W. McRae, Atlanta; vice-presidents, George S. Brown, Birmingham; J. Shelton Horsley, Richmond; secretary, W. D. Haggard, Nashville; treasurer, Charles M. Rosser, Dallas; council, George Ben Johnston, Richmond; L. McLane Tiffany, Baltimore; Lewis S. McMurtry, Louisville; J. Wesley Bovée, Washington City; Richard Douglas, Nashville; chairman committee of arrangements, John D. S. Davis, Birmingham.

At this meeting the monument erected to the memory of Dr. William E. B. Davis will be unveiled with ceremonies as follows: address of presentation, Dr. C. M. Rosser, Dallas, Texas; address of acceptance on behalf of state of Alabama, Governor R. M. Cunningham, M.D.; address of acceptance on behalf of the city of Birmingham, Hon. W. M. Drennan.

A list of thirty-six papers appears on the preliminary program, which cover interesting topics in surgery and gynecology.

---

The Pan-American Medical Congress will be held at Panama, January 2-6, 1905. Dr. Rudolph Matas, secretary of section of general surgery for the United States, asks those who wish to contribute papers to send title to him at No. 2255 Saint Charles avenue, New Orleans. He also announces that the United Fruit Company's agents are offering as a special inducement to American "congresistas" a reduction of the regular fare for the roundtrip from New Orleans to the Isthmus to $50.00, that is, $25.00 each way. The steamers leave New Orleans every Friday: the last steamer to leave New Orleans in time for the opening of the congress will sail on December 30, 1904, at 11 a. m. It takes about four and one-half days to reach Colon and seven days on the return trip on account of a stopover at Port Limon, where ample opportunity is given to tourists to visit San Jose, the beautiful capital of Costa Rica,—"the Paris of central America,"—where the most picturesque tropical scenery can be seen at this season, under the most favorable conditions.

---

The Harvard Medical Society of New York held a meeting in Hosack Hall, New York Academy of Medicine, November 26, 1904, at 8.30 p. m. An address by Professor Theobald Smith, of Harvard University, upon "The place of research in the university medical school," was the feature of the session. The president is Augustus S. Knight, and the secretary is J. Hilton Waterman, 50 West Fifty-first street, New York, formerly of Buffalo.

---

The Buffalo Academy of Medicine held meetings during the month of November as follows:

*Section on Surgery.*—Tuesday evening, November 1. Program: Classic art in medicine and surgery; illustrated by stereopticon, Roswell Park.

*Section on Medicine.*—Tuesday (Election) evening, November 8. Program: (a) Problems in dietetics, W. Gilman Thompson, professor of medicine in the Cornell University Medical College, New York city; discussion opened by Charles G. Stockton and Frederick C. Busch; (b) The value of certain procedures in the treatment of pneumonia and its complications, De Lancey Rochester; (c) The diagnosis of kidney function, Harvey R. Gaylord.

*Section on Pathology.*—Tuesday evening, November 15. Program: (a) A case of myeloma; (b) Demonstration of an unusual blood specimen of malaria, Charles Bentz; specimens will be presented by H. U. Williams, Eugene A. Smith and Marshall Clinton.

*Section on Obstetrics and Gynecology.*—Tuesday evening, November 22. Program: Alexander's operation; its immediate and remote results, James E. King; discussion by H. E. Hayd and S. Y. Howell.

